# Theoretical Study on the High HER/OER Electrocatalytic Activities of 2D GeSi, SnSi, and SnGe Monolayers and Further Improvement by Imposing Biaxial Strain or Doping Heteroatoms

**DOI:** 10.3390/molecules27165092

**Published:** 2022-08-10

**Authors:** Cuimei Li, Guangtao Yu, Xiaopeng Shen, Ying Li, Wei Chen

**Affiliations:** 1 Laboratory of Theoretical and Computational Chemistry, Institute of Theoretical Chemistry, Jilin University, Changchun 130023, China; 2 Engineering Research Center of Industrial Biocatalysis, Fujian Province Higher Education Institutes, Fujian Provincial Key Laboratory of Advanced Materials Oriented Chemical Engineering, Fujian-Taiwan Science and Technology Cooperation Base of Biomedical Materials and Tissue Engineering, College of Chemistry and Materials Science, Fujian Normal University, Fuzhou 350007, China; 3 Department of Chemistry and Chemical Engineering, Institute of Micro and Nano Functional Materials, Yancheng Institute of Technology, Yancheng 224051, China; 4 Fujian Provincial Key Laboratory of Theoretical and Computational Chemistry, Xiamen University, Xiamen 361005, China

**Keywords:** 2D materials (GeSi, SnSi, SnGe), oxygen evolution reaction (OER), hydrogen evolution reaction (HER), biaxial strain, heteroatom doping

## Abstract

Under the DFT calculations, two-dimensional (2D) GeSi, SnSi, and SnGe monolayers, considered as the structural analogues of famous graphene, are confirmed to be dynamically, mechanically and thermodynamically stable, and all of them can also possess good conductivity. Furthermore, we systematically investigate their electrocatalytic activities in overall water splitting. The SnSi monolayer can show good HER catalytic activity, while the SnGe monolayer can display remarkable OER catalytic activity. In particular, the GeSi monolayer can even exhibit excellent bifunctional HER/OER electrocatalytic activities. In addition, applying the biaxial strain or doping heteroatoms (especially P atom) can be regarded as the effective strategies to further improve the HER activities of these three 2D monolayers. The doped GeSi and SnSi systems can usually exhibit higher HER activity than the doped SnGe systems. The correlative catalytic mechanisms are also analyzed. This work could open up a new avenue for the development of non-noble-metal-based HER/OER electrocatalysts.

## 1. Introduction

Renewable energy storage and conversion technologies can play a key role in effectively alleviating the global environmental pollution and energy crisis [1,2,3,4,5]. Among them, electrochemical overall water splitting has received great attention, including two half-cell reaction processes of hydrogen evolution reaction (HER) and oxygen evolution reaction (OER). However, the HER and OER processes can only be carried out with the help of high-performance catalysts because their kinetic rates are too slow. At present, noble metals Pt and RuO_2_/IrO_2_ have been demonstrated to be the most efficient catalysts for HER and OER, respectively [6,7,8]. However, the rare reserves and high price of these metals hinder their large-scale application. Therefore, it is of great significance to develop the noble-metal-free HER and OER electrocatalysts with high catalytic activity.

Compared with conventional bulk materials, two-dimensional (2D) materials have aroused great interest in electrocatalysis because the unique planar structure with atomic thickness can bring many advantages, such as offering large specific area and a large number of active sites, permitting them as the optimum platform to easily couple with other materials, and promoting them as easily activated and optimized to achieve higher electrocatalytic activity through imposing strain or introducing defects and heteroatoms.

Among electrocatalysts, 2D monofunctional electrocatalysts, which can work for one half-reaction HER or OER, have been intensively investigated, such as carbon materials [9,10], transition metal-based materials [11,12], metal–organic frameworks (MOFs) [13,14], and so on. In addition, 2D bifunctional electrocatalysts catalyzing two different reactions have also attracted the interest of researchers, in view of more advantages. For example, they can have low overpotentials under the same and optimal reaction conditions, which can reduce the cost and greatly simplify the technological process [15]. It is reported that some 2D materials can be regarded as the bifunctional HER/OER electrocatalysts, such as transition-metal carbides [3], transition-metal borides [16], MOFs [17,18,19], and covalent organic frameworks (COFs) [20] etc.

Graphene consisting of six-membered carbon rings can be considered as one of the most important members in the 2D material family [21]. It is well known that the pristine graphene can show electrochemical inertness [22,23]. In view of this, great efforts have been made to improve the catalytic performance of HER or OER for 2D graphene through different strategies [24,25,26,27,28,29,30,31]. For instance, the B-doped graphene can serve as the efficient HER electrocatalyst [24]. The transition metal atoms anchored on the N-doped graphene can also be used as a good HER electrocatalyst [25]. In addition, the N-doped graphene can be regarded as a cost-effective electrocatalyst for OER [26]. The P-doped graphene can also have good OER activity [27]. In particular, some heteroatom-doped graphene systems can even exhibit bifunctional activity for HER and OER [28,29,30]. In addition, graphene defects trapping Ni atom species can also display bifunctional HER/OER catalytic activities [31].

Series of 2D binary GeSi, SnSi, and SnGe monolayers are composed of Si/Ge/Sn atoms with the larger atomic radius in the same main group as carbon, all of which can be considered as the analogue of famous graphene. According to previous reports [32,33], all of them are dynamically stable, and they have very small band gap or metallic behavior, indicating good conductivity. In addition, compared with graphene, 2D GeSi, SnSi, and SnGe systems can reduce π bonding and form mixed sp^2^-sp^3^ orbitals, leading to the low-buckled structures, which will be favorable for the attack of reactants. In addition, the charge transfer between two adjacent atoms with different electronegativity in each monolayer can effectively adjust the electron density on the relevant atoms. All of these could be advantageous for the HER or OER catalytic activity of the material system. However, to the best of our knowledge, the correlative electrocatalytic performance of the 2D GeSi, SnSi, and SnGe monolayers has never been reported, in spite of the importance and significance. It is worth mentioning that recently the Na-, K-, and Ca-functionalized 2D GeSi systems could be useful as hydrogen storage media for mobile applications [34]. In addition, 2D SnSi and SnGe systems have great potential as a new generation of electrode materials [35]. In this study, we would like to investigate the HER or OER electrocatalytic activities of all three of these 2D GeSi, SnSi, and SnGe monolayers, and it is highly expected that they can exhibit good electrocatalytic performance and even show bifunctional electrocatalytic activity concerning HER/OER. Furthermore, we also proposed two effective ways through applying biaxial strain and doping heteroatoms to further improve their electrocatalytic activities.

## 2. Results and Discussion

### 2.1. The Geometries, Stabilities, and Electronic Properties of 2D GeSi, SnSi, and SnGe Monolayers

**Figure 1:**
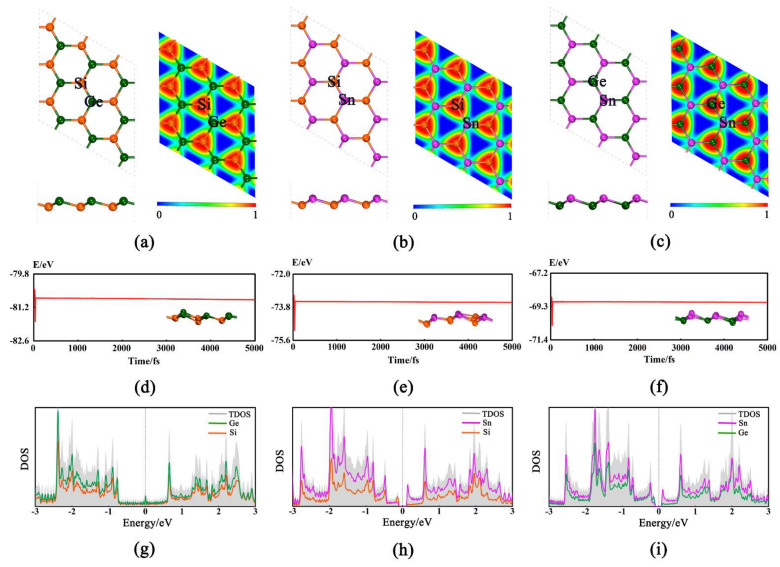
Optimized structures (top and side views) and electron location function (ELF) for GeSi (**a**), SnSi (**b**), and SnGe (**c**). The Si, Ge, and Sn atoms are represented by orange, dark green, and purple balls, respectively. The trajectory of ab initio molecular dynamic simulation at 500 K (the final corresponding structure snapshot is given in inset) of GeSi (**d**), SnSi (**e**), and SnGe (**f**). The density of states (DOS) for GeSi (**g**), SnSi (**h**), and SnGe (**i**).

The 2D GeSi, SnSi, and SnGe monolayers can have the buckled honeycomb structures (Figure 1a–c), all of which belong to *P3M1* space group and have been demonstrated to be dynamically stable [33]. Their lattice parameters are a = b = 3.926 Å, a = b = 4.234 Å, and a = b = 4.305 Å (Table S1), respectively. In addition, all the calculated bond lengths of Ge-Si, Sn-Si, and Sn-Ge are 2.345, 2.547, and 2.610 Å, respectively, which are basically consistent with the previous results [32,35,36].

Based on the optimized structures, the thermal stabilities of three 2D monolayers are examined through performing AIMD simulations [37] at 500 K. As shown in Figure 1d–f, the total energy can fluctuate near the equilibrium value and the structure can be well retained, confirming their good thermal stabilities. Moreover, the mechanical stability for three pristine monolayers is also evaluated by considering the linear elastic constants (Table S1). As we know, the necessary and sufficient conditions [38] for a mechanically stable 2D hexagonal structure are C_11_ > 0, C_44_ > 0 and C_11_ − C_12_ > 0. Our computed elastic constants can satisfy the above conditions, confirming that they are mechanically stable, namely, C_11_ = 59.41 N.m^−1^, C_12_ = 17.71 N.m^−1^, C_44_ = 20.92 N.m^−1^ for GeSi, C_11_ = 15.76 N.m^−1^, C_12_ = 10.29 N.m^−1^, C_44_ = 12.06 N.m^−1^ for SnSi, and C_11_ = 31.58 N.m^−1^, C_12_ = 17.05 N.m^−1^, and C_44_ = 8.10 N.m^−1^ for SnGe, respectively. In addition, the electron location function (ELF) [39] was used to analyze the nature of the correlative Ge-Si, Sn-Si, and Sn-Ge bonds (Figure 1a–c). The large ELF value (about 0.85) indicates that the valence electrons among two adjacent atoms can be shared with each other and the covalent Ge-Si, Sn-Si, or Ge-Sn bond can be formed, which can be responsible for the high stability. Moreover, our computed density of states (DOS) shows that all three of these 2D monolayers can possess the metallic behavior or very small band gaps (Figure 1g–i and Table S1), indicating their good conductivity. Obviously, all the above advantages can be beneficial to the HER/OER electrocatalytic performance of the studied systems, which prompts us to carry out the following relevant research.

### 2.2. The HER/OER Catalytic Activities of 2D Pristine GeSi, SnSi, and SnGe Monolayers

In this section, we present the detailed investigations on the HER and OER catalytic activities of all three 2D GeSi, SnSi, and SnGe monolayers. Initially, their HER catalytic activities are evaluated by calculating the free energy of H* (ΔG_H*_). It is well known that the HER activity over a given system can be closely correlated to the adsorption energy of a single H atom on the system, and thus ΔG_H*_ can be used as a reliable indicator to evaluate the HER activity on the system [40,41]. Usually, a smaller absolute value of ∆G_H__∗_ means better HER activity. It is worth mentioning that the descriptor ΔG_H*_ has been used extensively for various systems that can catalyze HER [9,10,42,43,44,45,46,47,48].

**Figure 2:**
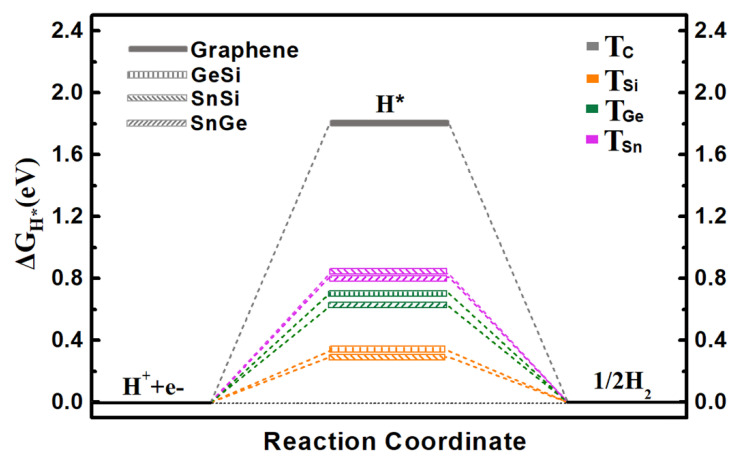
The calculated free-energy diagram of HER on the pristine graphene, GeSi, SnSi, and SnGe structures at equilibrium potential for different adsorption sites.

Specifically, we calculate the ΔG_H*_ values on three 2D monolayers by considering all possible adsorption sites, where the two top sites over Ge/Si atoms (T_Ge_ and T_Si_), Sn/Si atoms (T_Sn_ and T_Si_), and Sn/Ge atoms (T_Sn_ and T_Ge_) can be ultimately obtained for the GeSi, SnSi, and SnGe systems, respectively (Figure 1a–c). The calculated ΔG_H*_ values of three monolayers including GeSi, SnSi, and SnGe are in the range of 0.293~0.826 eV (Figure 2 and Table S2), all of which can be much smaller than that of graphene (1.804 eV), indicating the change of HER catalytic activity towards a good trend. Specifically, all the Si sites can uniformly have small ΔG_H*_ values as 0.332 eV for GeSi and 0.293 eV for SnSi, respectively, meaning that the Si sites on these two pristine systems can exhibit good HER activity. Different from the Si sites, the T_Ge_ site (0.704 eV) on GeSi and the T_Sn_ site (0.826 eV) on SnSi can have large ΔG_H*_ values, reflecting the relatively poor HER activities at these two sites. Clearly, the Si sites can serve as the active sites on the two 2D GeSi and SnSi monolayers. Comparatively, the calculated ΔG_H*_ values of T_Ge_ and T_Sn_ on the 2D SnGe system are 0.620 and 0.819 eV, respectively. Such a large ΔG_H*_ value suggests the relatively inert HER activity. Obviously, among these three 2D monolayers, the GeSi and SnSi systems can exhibit good HER catalytic activity, where Si sites can be responsible for their catalytic performance.

Subsequently, we investigate the OER catalytic activities of these three 2D monolayers. As we know, the OER can be considered a four-step process. Usually, the energy barriers of two neighboring elementary steps should be 1.23 V for the ideal OER electrocatalysts, and the overpotential (η) is zero. Nevertheless, the distance of two adjacent elementary steps is not always equal, which results in different η_OER_ determined by the largest energy difference.

**Figure 3:**
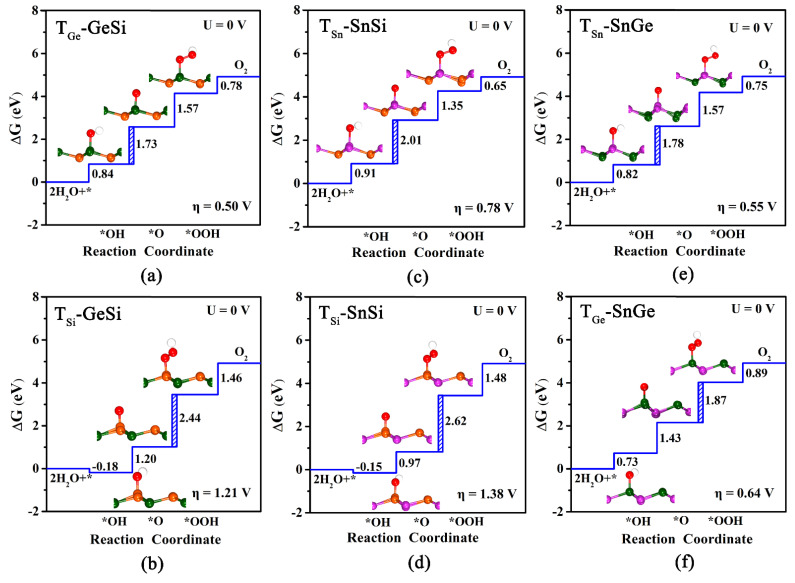
The calculated free energy diagram at T_Ge_/T_Si_, T_Sn_/T_Si_ and T_Sn_/T_Ge_ sites, and the corresponding configurations of adsorbed intermediates on pure GeSi (**a**,**b**), SnSi (**c**,**d**), and SnGe (**e**,**f**) monolayers for OER under U = 0 V. The blue dashed area represents the rate-limiting step for OER.

In this study, we investigate the OER catalytic activities of three 2D monolayers by estimating the overpotential η_OER_ values, as shown in Figure 3a–f. Finally, two adsorption sites can be obtained for each monolayer system, namely T_Ge_ and T_Si_ for GeSi (Figure 3a,b), T_Sn_ and T_Si_ for SnSi (Figure 3c,d), T_Sn_ and T_Ge_ for SnGe (Figure 3e,f), respectively. We can find that all these obtained adsorption sites can have lower overpotential η_OER_, compared with the pristine graphene (1.43 V). To be specific, our computed results reveal that T_Ge_ (0.50 V) of GeSi, T_Sn_ (0.55 V) of SnGe, and T_Sn_ (0.78 V) of SnSi can be the optimal active sites with a lower overpotential on their respective monolayers. For these three active sites, the potential-determining step can uniformly be the second electron transfer step, that is, from OH* to *O. In addition, we can also find that the calculated η_OER_ values for T_Ge_-GeSi (0.50 V) and T_Sn_-SnGe (0.55 V) are much lower than T_Sn_-SnSi (0.78 V), suggesting higher OER catalytic activity. In particular, it is worth mentioning that the η_OER_ values of T_Ge_-GeSi and T_Sn_-SnGe are even comparable to the state-of-the-art catalyst IrO_2_ (η_OER_ = 0.56 V) [49], reflecting their excellent OER catalytic activities.

Comparatively, the third electron transfer step, from O* to *OOH, becomes the most energy-consuming for the other sites. Specifically, the calculated η_OER_ value of T_Ge_ on SnGe is 0.64 V, indicating good OER catalytic activity. However, the high overpotential η_OER_ for T_Si_-GeSi (1.21 V) and T_Si_-SnSi (1.38 V) can mean poor OER catalytic activities at the relevant Si sites, which is different from the case of HER. Clearly, the GeSi and SnGe systems among these three 2D monolayers can exhibit considerably high OER catalytic activity, where the Ge site and the Sn site can serve as their corresponding most active sites.

Overall, compared with graphene, the HER and OER catalytic activities of all the 2D GeSi, SnSi, and SnGe binary monolayers can change uniformly towards a good trend. Both GeSi and SnSi systems can exhibit good HER activity, while both GeSi and SnGe systems can display excellent OER activity. In particular, the 2D GeSi monolayer can be used as bifunctional HER and OER electrocatalyst. Their high HER/OER catalytic activities can be mainly attributed to the following aspects: (1) Compared with the analogous graphene, all three of these 2D monolayers composed of Si/Ge/Sn atoms with larger atomic radius can uniformly possess relatively weak π-π bonding, resulting in the formation of low buckled structures with mixed sp^2^-sp^3^ orbitals, which is advantageous for the attacking of reactants. (2) The calculated Mulliken charges reveal that the electron transfer process (ca. 0.05~0.23 |e|) can be observed between two adjacent atoms in these three 2D systems due to the different electronegativity between them. This can effectively activate the relevant atoms by adjusting their electron densities.

### 2.3. Two Effective Strategies to Improve HER Catalytic Activities of 2D Pristine GeSi, SnSi, and SnGe Monolayers

From the above discussions, we can know that both the GeSi and SnGe systems can possess excellent OER activity. Comparatively, the catalytic activity of HER needs to be further improved in view of the fact that the calculated ∆G_H__∗_ values for all the three materials are not very close to zero. The catalytic activity of the catalyst can be improved through using different strategies [50,51]. In this section, two effective ways through applying strain and doping heteroatoms are proposed to enhance the HER activities of these 2D pristine GeSi, SnSi, and SnGe systems.

#### 2.3.1. Applying Biaxial Strain to Boost the HER Catalytic Activities of GeSi, SnSi, and SnGe Systems

Initially, we propose an effective strategy through applying the biaxial strain to optimize the electronic structure and improve the HER activity for the 2D GeSi, SnSi, and SnGe monolayers considering that these three buckled structures could bear a certain range of stress.

**Figure 4:**
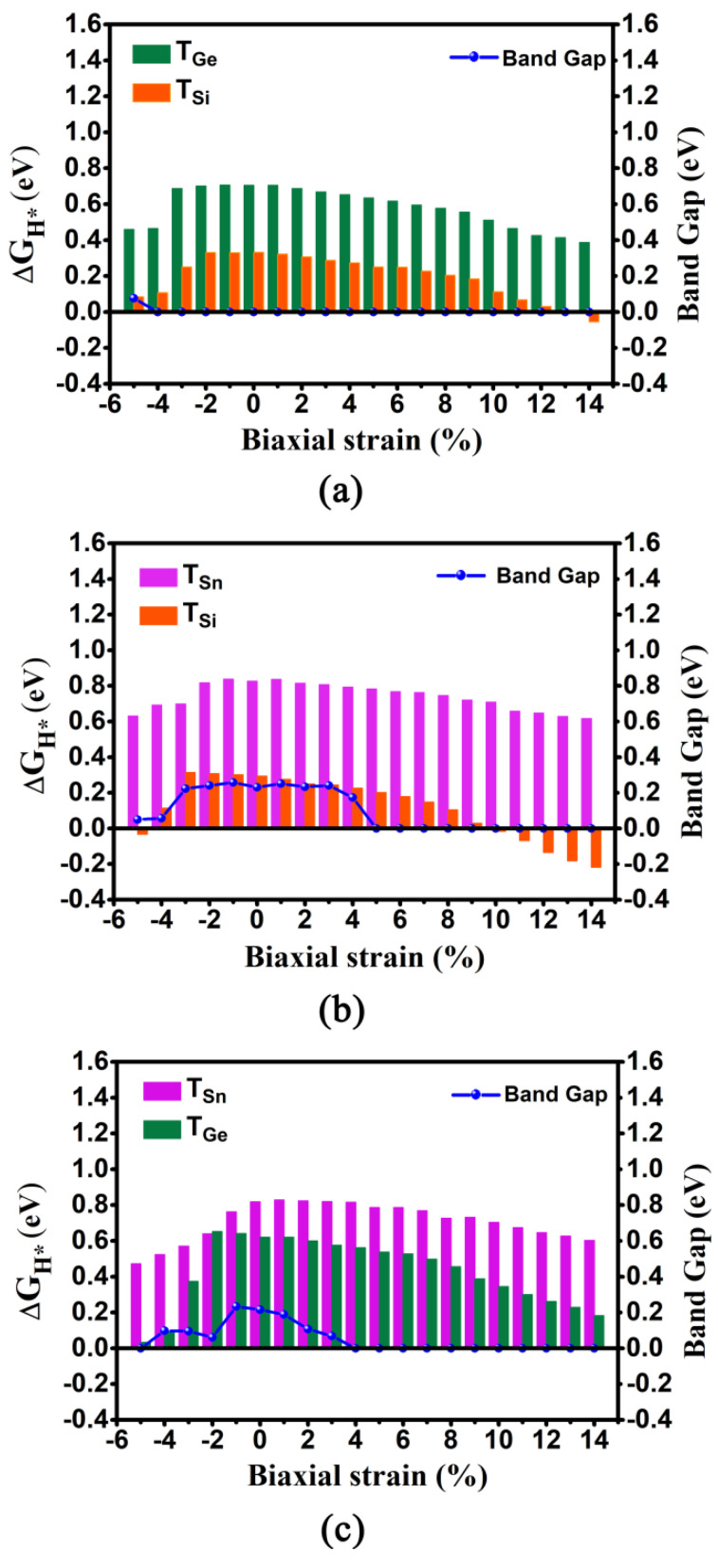
The computed ΔG_H*_ values at the T_Ge_/T_Si_, T_Sn_/T_Si_, and T_Sn_/T_Ge_ sites and band gaps, as a function of compressive and tensile biaxial strains for the 2D GeSi (**a**), SnSi (**b**), and SnGe (**c**) systems.

We calculated the ΔG_H*_ values and corresponding DOSs of these three 2D monolayers under different biaxial strain in the range of −5%~14% (Figure 4 and Figure S1 and Table S3). As for the GeSi system, when imposing the biaxial strain, the absolute |ΔG_H*_| values for T_Si_ and T_Ge_ sites can uniformly show a decreasing trend, regardless of biaxial compressive or tensile strain. Specifically, it can be found that the ΔG_H*_ values can decrease sharply with the increase of the biaxial compressive strain from 0 to −5%, while the ΔG_H*_ values gradually decrease with the increase of biaxial tensile strain from 0 to 14% (Figure 4a and Table S3). In particular, when increasing the biaxial tensile strain from 5 to 14%, very small ΔG_H*_ values ranging from 0.250 to −0.054 eV can be observed at the T_Si_ site, indicating considerably high HER activity, and the ΔG_H*_ values of the T_Ge_ site can be decreased from 0.634 to 0.387 eV, indicating the improvement of HER activity at this site. In contrast, when increasing the biaxial compressive strain from −3% to −5%, very small ΔG_H*_ values varying from 0.250 to 0.084 eV can also be observed at the T_Si_ site, and the ΔG_H*_ values of the T_Ge_ site can be reduced from 0.687 to 0.460 eV. Moreover, independent of biaxial compressive or tensile strain, the metallic behavior can be sustained, except that the GeSi system has only a near-zero band gap of 0.076 eV under the −5% biaxial compressive strain (Figure 4a and Table S4), which indicates good conductivity. Clearly, applying biaxial tensile strain of 5%~14% and biaxial compressive strain of −3%~−5% can endow the 2D GeSi monolayer with considerably high HER activity, in view of very small ΔG_H*_ values at the Si sites and good conductivity (Figure 4 and Figure S1a and Tables S3 and S4).

The similar decreasing trend of ΔG_H*_ values at the T_Sn_ and T_Si_ sites can be also observed on the 2D SnSi system (Figure 4b and Table S3). Specifically, it can be found that the Sn sites are not sensitive to the applied biaxial strain in view of large ΔG_H*_ values, in the range of 0.617~0.838 eV. Differentially, the ΔG_H*_ values at the Si sites can be very small, in the range of −0.032~0.114 eV under the compressive strain of −4%~−5% and in the range of −0.217~0.250 eV under the tensile strain of 2%~14%, respectively. In particular, imposing 10% biaxial tensile strain can bring the highest HER catalytic activity, in view of the optimum ΔG_H*_ value at the T_Si_ (−0.015 eV). Clearly, the application of biaxial strain can effectively enhance the HER catalytic activity of the Si site on the SnSi system. In addition, no matter whether imposing the biaxial compressive or tensile strain, the band gap of SnSi can be reduced (Figure 4b). In particular, when the compressive strain is in the range of −4%~−5% or the tensile strain is larger than 5%, near-zero band gap and even the metallic behaviors can be observed, indicating good conductivity (Figure S1b and Table S4).Obviously, applying the biaxial tensile strain of 5%~14%and compressive strain of −4%~−5% can effectively improve HER performance of the 2D SnSi system by enhancing the catalytic activity of Si sites, simultaneously accompanied by good conductivity.

As for the SnGe system, when applying a series of biaxial strains ranging from −5% to 14%, the ΔG_H*_ values of T_Ge_ and T_Sn_ sites can be in the range of 0.033~0.652 eV and 0.473~0.829 eV, respectively (Table S3). It can be found that the ΔG_H*_ value at the T_Ge_ site can be uniformly decreased, regardless of imposing the biaxial compressive or tensile strain (Figure 4c and Table S3). In contrast, the application of the biaxial compressive strain or biaxial tensile strain can merely have a small effect on the ΔG_H*_ value at the T_Sn_ site. In addition, imposing the biaxial tensile strain can reduce the band gap of the 2D SnGe system, and the turning point appears at the tensile strain of 4% where metallic behavior can be observed (Figure 4c and Table S4). Evidently, applying the biaxial tensile strain of 13%~14% or biaxial compressive strain of −5% can bring high HER catalytic activity at the T_Ge_ site, due to their very small ΔG_H*_ values (0.033~0.228 eV) and good conductivity (Figure S1c and Tables S3 and S4).

Overall, applying the biaxial strain can be considered as an effective way to enhance the HER catalytic activity for 2D GeSi, SnSi, and SnGe monolayers through optimizing ΔG_H*_ value and electronic properties, especially for the GeSi and SnSi systems containing Si.

#### 2.3.2. Doping Heteroatoms to Enhance the HER Activities of GeSi, SnSi, and SnGe Systems

In addition to the application of biaxial strain, doping heteroatoms is also proposed as another strategy to boost the HER activity of 2D GeSi, SnSi, and SnGe systems. In this study, we choose the relevant atoms with similar atomic radiuses in the same period to replace the target atoms, that is, P and S are used as dopants for Si atom, As and Se are used as dopants for Ge atom, and Sb and Te are used as dopants for Sn atom. In addition, we also replace the target atoms Sn and Ge with P atoms.

**Figure 5:**
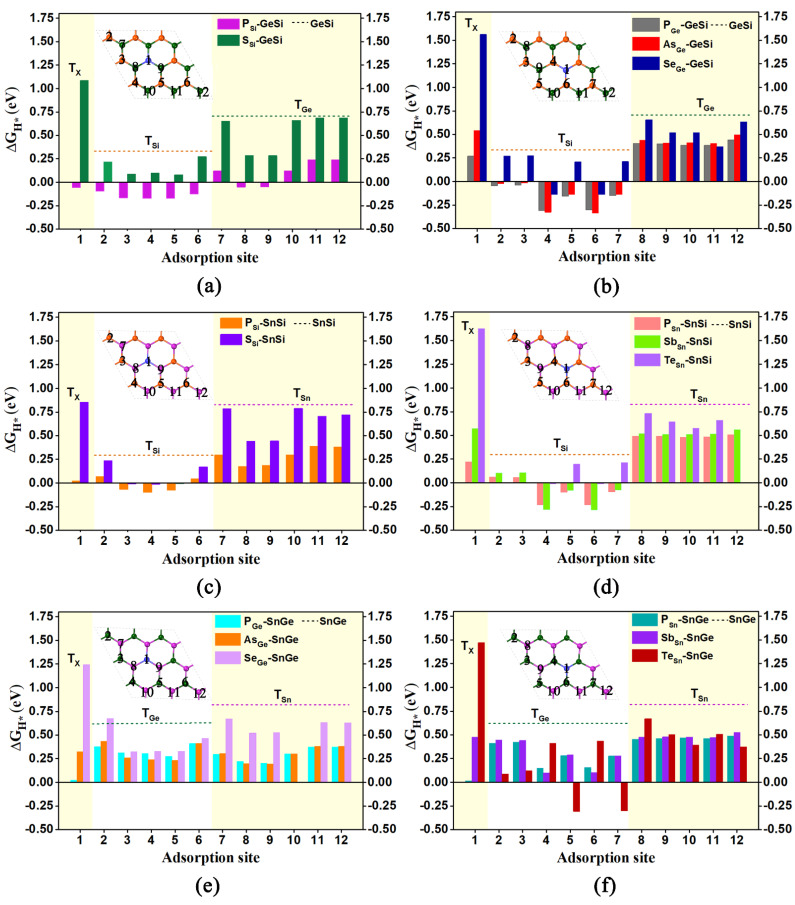
The doped GeSi, SnSi, and SnGe structures with the foreign atoms where all the obtained adsorption sites are marked, as well as the computed free-energy diagram of HER for doped GeSi (**a**,**b**), SnSi (**c**,**d**), and SnGe (**e**,**f**) systems for different adsorption sites. The orange, dark green, and purple dashed lines represent the ΔG_H*_ of the T_Si_, T_Ge_, and T_Sn_ site on these pure systems, respectively.

Ultimately, a total of 16 doped systems were obtained by doping the above atoms into 2D GeSi, SnSi, and SnGe monolayers. For convenience, all these doped systems can be represented as X-GeSi, X-SnSi, and X-SnGe, respectively. For example, the doped GeSi systems with X at the Si site can be named X_Si_-GeSi, where X represents the dopant atom (X= P or S), and the subscript represents the substitution site. In addition, adding the Arabic number *n* after the top site (T) can distinguish all possible adsorption sites in the corresponding systems, as shown in Figure 5a–f.

The computed results reveal that all the doped GeSi, SnGe, and SnSi systems can usually maintain the original geometric features, in which S-, Se-, or Te-doping can merely cause a small local deformation, in contrast to other dopants used, as illustrated in Figures S2–S4. The computed DOS results show that the metallic behavior or small band gap can be observed for all these doped systems, which indicates that good conductivity can still be maintained (Figures S2–S4).

Based on the optimized geometry structures, we investigated the HER catalytic activities of all the doped GeSi, SnSi, and SnGe systems. More correlative details have been provided in supplementary information (Tables S5–S7). Initially, we focus on the HER catalytic activities of five GeSi systems doped with P and S at the Si site (Figure 5a) as well as doped with P, As, and Se at the Ge sites (Figure 5b), respectively. All of them can exhibit higher HER catalytic activity than the pristine one, as indicated by the smaller ΔG_H*_ values of some relevant adsorption sites (Figure 5a,b and Table S5). As for the substitution of the Si site, doping P can endow all the top sites over Si/Ge atoms and the doping site with very small ΔG_H*_ values, in the range of −0.171 eV~0.240 eV, inducing considerably high HER catalytic activity. Comparatively, only the vast majority of Si sites can have the small ΔG_H*_ values varying from 0.079 eV to 0.217 eV for the S-doped GeSi. Obviously, doping P at the Si site can bring higher HER catalytic activity on the GeSi monolayer than doping S at the Si site in view of the formation of more highly active sites. Similarly, doping P at the Ge site can also endow the GeSi monolayer with better HER catalytic activity than doping As or Se at the Ge site in view of the smaller absolute ΔG_H*_ values. In addition, it can be found that the Si sites can uniformly act as the active site on these three doped GeSi systems with the dopant at the Ge site.

Obviously, independent of the dopants, doping the heteroatoms including P, S, As, and Se can effectively improve the HER activity of the 2D GeSi monolayer. Among them, P can be regarded as the best dopant, since more highly active sites with the smaller ΔG_H*_ values can be produced upon doping P. In addition, doping P at the Si site can induce higher HER activity than doping P at the Ge site in view of the fact that both the Ge- and Si sites of the former can be used as the active sites, but only Si sites can be used as the active sites of the latter.

Subsequently, we examined the HER catalytic activities of SnSi systems doped with P and S at the Si site (Figure 5c) as well as doped with P, Sb, and Te at the Sn site (Figure 5d), respectively. Similarly, all these doped SnSi structures can present higher HER catalytic activity than the pristine one, as revealed by the smaller ΔG_H*_ values of some relevant adsorption sites (Figure 5c,d and Table S6). Specifically, as for the substitution of the Si site, doping P can effectively enhance the HER performance of the 2D SnSi monolayer, where all the Si sites and some Sn sites as well as the doping sites can possess considerably high HER activity, in view of the much smaller ΔG_H*_ values with the range of −0.099 eV~0.184 eV. Comparatively, when doping S at the Si site, only all the Si sites can have the much smaller ΔG_H*_ values ranging from −0.016 eV to 0.235 eV. Clearly, doping P at the Si site can also bring higher HER activity on the 2D SnSi monolayer than doping S atom at the Si site due to the emergence of more highly active sites. A similar situation can be observed in the SnSi systems doped with P, Sb, and Te at the Sn sites; that is, doping of P can more effectively improve the HER activity of the SnSi monolayer than doping of Sb or Te, and the Si sites can uniformly play a crucial role in determining their high HER activities.

Clearly, doping the heteroatoms involving P, S, Sb, and Te can effectively enhance the HER catalytic activity of the 2D SnSi system, in which the Si sites can make an important contribution. In particular, the P atom can be considered as the most suitable dopant to improve the HER activity on the SnSi monolayer and doping P at the Si site can bring higher HER catalytic activity than at the Sn site, in view of the formation of more active sites.

Finally, we also performed the ΔG_H*_ calculations for evaluating the HER activities of the related SnGe systems doped with P, As, and Se at the Ge site (Figure 5d) as well as doped with P, Sb, and Te at Sn site (Figure 5e), respectively. Our computed results reveal that all these doped SnGe systems can present better HER catalytic activity than the pristine one (Figure 5d,e and Table S7). In addition, it can be found that compared with other dopants, doping P or As atoms at the Ge site can more effectively improve the HER catalytic activity of the 2D SnGe monolayer.

**Figure 6:**
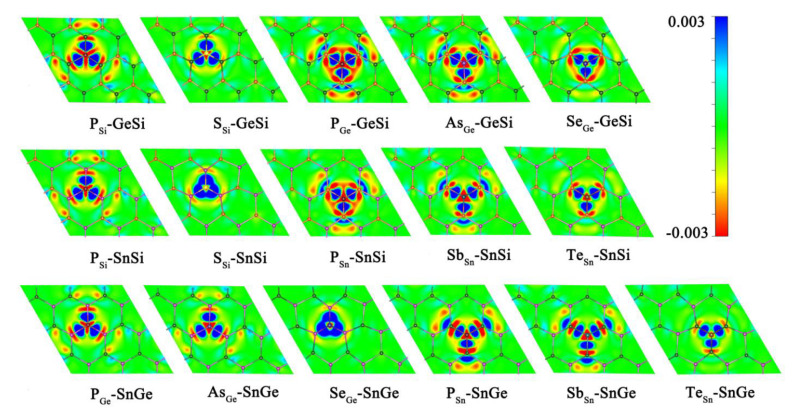
The charge density difference Δρ values for the doped GeSi, SnSi, and SnGe systems with the red and blue areas meaning low and high electron densities, respectively.

Obviously, doping the relevant heteroatoms can be considered as an effective strategy to enhance the HER activity for 2D GeSi, SnSi, and SnGe monolayers. The doped GeSi and SnSi systems can usually exhibit higher HER catalytic activity than the doped SnGe systems. The improvement of the HER catalytic activity can be attributed to the fact that doping of the related foreign atoms can induce the evident electron transfer process between dopant X and the GeSi/SnSi/SnGe part in these doped systems, further activating the correlative Si, Ge, and Sn atoms (Figure 6).

Moreover, all the P-doped GeSi, SnSi, and SnGe monolayers can usually possess higher HER catalytic activity, and the P-doped GeSi system at the Si site among them can exhibit the highest HER catalytic activity, where all the top sites on the material surface can uniformly serve as highly active sites. Therefore, P atom can be regarded as the most effective dopant to enhance HER activity on these 2D monolayers. We can find that in addition to activating the relevant Si/Ge/Sn atoms more effectively, doping P can also bring the doping sites T_P_ with excellent HER activity on the surfaces of GeSi, SnSi, and SnGe, where the corresponding |ΔG_H*_| values are very small in the range of 0.017~0.269 eV. Herein, we have carried out a detailed mechanism analysis of the high HER activity of the doping site T_P_. As we know, when the adsorbed H* interacts with the P site, one valence electron of H atom and the lone pair of P atom can form one fully filled bonding orbital (σ_P-H_) and one partially filled antibonding orbital (σ*_P-H_). Therefore, the strength of the interaction between H and P atoms can be dominated by the occupancy of σ*_P-H_. Our calculated Mulliken charge shows that an effective electron transfer process (ca. −0.46 ~ −0.18 |e|) can be observed from the related adjacent atoms to the dopant P, leading to the increase of the occupancy of σ*_P-H_. This can effectively weaken the bonding between H and P atoms, and make the T_P_ site have an appropriate adsorption state of H*, thereby having high HER catalytic activity. The weakened P-H bonding can also be confirmed by the corresponding structural parameters; that is, the calculated P-H bond lengths are in the range of 1.433–1.438 Å, which are longer than the single bond of P-H (1.421 Å).

## 3. Computational Methods

The generalized gradient approximation of the Perdew–Burke–Ernzerhof function (GGA/PBE) [52] was used to perform all the density functional theory (DFT) computations ofthe studied systems within the frame of the Vienna Ab Initial Simulation Package (VASP) [53,54]. A 3 × 3 × 1 supercell was used to guarantee the accuracy and efficiency of calculation results, and a vacuum region of 20 Å along the *z*-direction was set to avoid the spurious interactions between adjacent units. The kinetic energy cutoff was set to 500 eV and a semi-empirical van der Waals correlation (vdW) proposed by Grimme (DFT-D2) was used to account for the dispersion interactions [55,56]. In addition, the Monkhorst–Pack grid k-points of 5 × 5 × 1 and 63 k-points were employed for the structural optimization and the density of states (DOSs) of our studied work, respectively. In addition, the convergence criterion of energy and force in calculations were set to 1.0 × 10^−4^ eV and 0.02 eV Å^−1^, respectively.

The HER catalytic activity was estimated using the Gibbs free energy change (ΔG_H*_), which can be defined by the following equation:

ΔG_H*_ = ΔE_H*_ + ∆ZPE − T∆S_H*_ (1)

in which ΔE_H*_ stands for the energy difference of hydrogen adsorption, and ΔZPE and ∆S denote the corresponding changes of zero point energy and entropy of H* adsorption, respectively.

The OER proceeding via the four electron-transfer steps can be written as follows:

H_2_O + * → *OH + H^+^ + e^−^ (2)

*OH → *O + H^+^ + e^−^ (3)

H_2_O + *O → *OOH + H^+^ + e^−^ (4)

*OOH → * + O_2_ + H^+^ + e^−^ (5)

The Gibbs free-energy change (ΔG) for each elemental step in the OER process can be obtained by the following formula [57]:

ΔG = ΔE+ ∆ZPE − T∆S + ΔG_U_ + ΔG_pH_ (6)

in which ΔE, ∆ZPE, and ∆S represent the energy difference of adsorption, the corresponding changes of zero point energy, and the entropy of adsorption, respectively. The values (∆ZPE − T∆S) for each elemental step refer to the standard table [58]. ΔG_U_ = −neU, in which U stands for the applied electrode potential, and e and n represent the charge transferred and the number of proton–electron transferred pairs, respectively. ΔG_pH_ = −*k*_B_TIn[H^+^] = pH × *k*_B_TIn10, where *k*_B_ is the Boltzmann constant. ΔG_1_ = ΔG_OH*_, ΔG_2_ = ΔG_O*_ − ΔG_OH*_, ΔG_3_ = ΔG_OOH*_ − ΔG_O*_, and ΔG_4_ = 4.92 − ΔG_OOH*_.

Additionally, the overpotential can be computed withthe following equations:

η_OER_ = max {ΔG_1_, ΔG_2_, ΔG_3_, ΔG_4_}/e − 1.23 (7)

where ΔG_i_ (i = 1–4) and 1.23 represent the Gibbs free-energy change for step (i) and the equilibrium potential, respectively.

## 4. Conclusions

In summary, these 2D GeSi, SnSi, and SnGe nanostructures can uniformly have high stability and good conductivity. Both the GeSi and SnSi monolayers containing Si can present good HER activity, where the Si sites can serve as the active sites. Both the GeSiand SnGe monolayers can exhibit excellent OER activity. In particular, the 2D GeSi monolayer can be used as bifunctional HER and OER electrocatalyst. Applying the biaxial strain can be regarded as an effective strategy to improve their HER catalytic activity through optimizing ΔG_H*_ value and electronic properties, especially for the GeSi and SnSi systems containing Si. In addition, doping the heteroatoms (especially P atom) can be considered as another effective strategy to improve HER catalytic performance of these three 2D systems. The doped GeSi and SnSi systems can usually exhibit higher HER catalytic activity than the doped SnGe systems, where Si sites can serve as the most active sites. Obviously, coupled with good conductivity, all three of these 2D monolayers can be considered as a promising new type of HER or OER catalyst. The reasons behind the high catalytic activity have also been analyzed in detail. This study can provide new ideas and help to design the highly efficient and nonprecious HER/OER electrocatalysts containing the IV main group elements.

## Data Availability

Not applicable.

## Supplementary Materials

The following supporting information can be downloaded at: https://www.mdpi.com/article/10.3390/molecules27165092/s1, Figure S1. The computed DOSs of GeSi (a), SnSi (b) and SnGe (c) under biaxial strains where the computed ΔG_H*_ values are ranging from −0.250 to 0.250 eV. For comparison, the computed DOSs of pristine GeSi, SnSi, and SnGe monolayer are plotted; Figures S2–S4. The top and side views of the doped GeSi, SnSi, and SnGe structures and their corresponding DOSs, respectively; Table S1. The space group, optimized lattice constant, the computed bond lengths d (Å), band gaps (eV) and elastic constants (N.m^−1^) for 2D GeSi, SnSi, and SnGe systems; Table S2. The computed ΔG_H*_ (eV) values for H* at the different adsorption sites (S_ad_) on the pristine GeSi, SnSi, and SnGe systems; Table S3. The computed ΔG_H*_ value at the T_Ge_/T_Si_, T_Sn_/T_Si_ and T_Sn_/T_Ge_ sites, as a function of compressive and tensile biaxial strains for the 2D GeSi, SnSi, and SnGe; Table S4. The calculated band gaps for the GeSi, SnSi, and SnGe as a function of compressive and tensible biaxial strains; Table S5. The calculated ΔG_H*_ values for the different adsorption sites on the X_Si_-GeSi and X_Ge_-GeSi (X = P, S As or Se) systems. Refer to Figure 5a,b for the different adsorption sites (S_ad_); Table S6. The calculated ΔG_H*_ values for the different adsorption sites on the X_Si_-SnSi and X_Sn_-SnSi (X = P, S, Sb or Te) systems. Refer to Figure 5c,d for the different adsorption sites (S_ad_); Table S7. The calculated ΔG_H*_ values for the different adsorption sites on the X_Ge_-SnGe and X_Sn_-SnGe(X = P, As, Se, Sb or Te) systems. Refer to Figure 5e,f for the different adsorption sites (S_ad_).

## Sample Availability

Samples are available from the authors.
