# Supplementary material for: Theoretical Study on the High HER/OER Electrocatalytic Activities of 2D GeSi, SnSi, and SnGe Monolayers and Further Improvement by Imposing Biaxial Strain or Doping Heteroatoms
Source: Molecules. 2022 Aug 10;27(16):5092. doi: 10.3390/molecules27165092 (PMC9415490; doi:10.3390/molecules27165092)
Supplement: Supplementary file 1 [file molecules-27-05092-s001.zip › molecules-1832949-supplementary.pdf]

Supplementary Information

Theoretical Study on the High HER/OER Electrocatalytic Activities of 2D GeSi, SnSi and SnGe Monolayers and Further Improvement by Imposing Biaxial Strain or Doping Heteroatoms

Cuimei Li[1], Guangtao Yu[1, 2*], Xiaopeng Shen[3], Ying Li[1*] and Wei Chen[1, 2, 4*]

[1]*Laboratory of Theoretical and Computational Chemistry, Institute of Theoretical Chemistry, Jilin University, Changchun 130023, China*

[2]*Engineering Research Center of Industrial Biocatalysis, Fujian Province Higher Education Institutes, Fujian Provincial Key Laboratory of Advanced Materials Oriented Chemical Engineering, Fujian-Taiwan Science and Technology Cooperation Base of Biomedical Materials and Tissue Engineering, College of Chemistry and Materials Science, Fujian Normal University, Fuzhou 350007, China*

[3] *Department of Chemistry and Chemical Engineering, Institute of Micro and Nano Functional Materials, Yancheng Institute of Technology, Yancheng, Jiangsu 224051, China*

[4]*Fujian Provincial Key Laboratory of Theoretical and Computational Chemistry, Xiamen University, Xiamen, 361 005, China*

*Corresponding authors

E-mail: yugt@fjnu.edu.cn (G.T. Yu), liyingedu@jlu.edu.cn (Y. Li) and chenwei@fjnu.edu.cn (W. Chen)

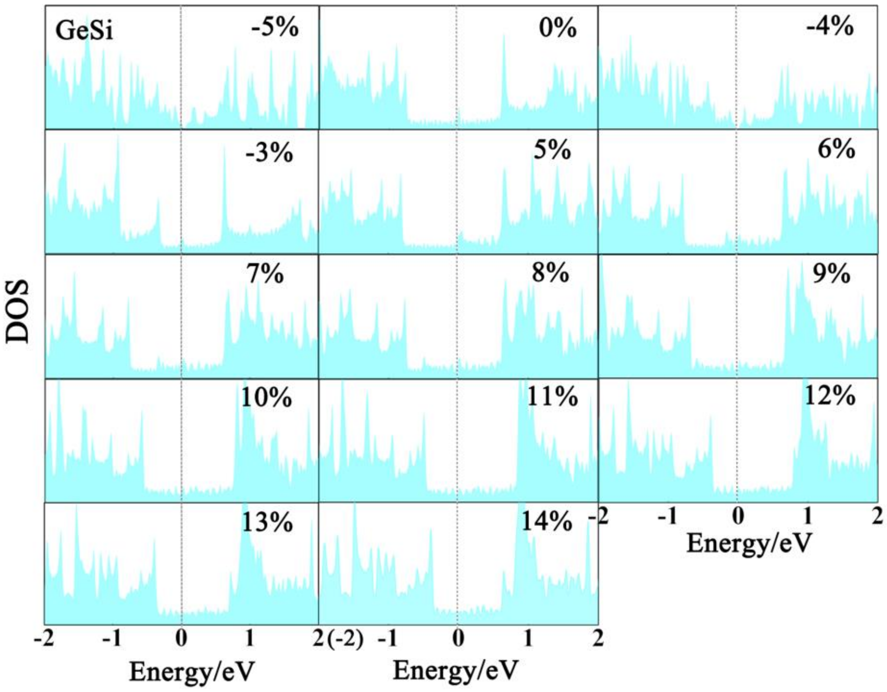
GeSi
-5%
0%
-4%
-3%
5%
6%
7%
8%
9%
10%
11%
12%
13%
14%
DOS
Energy/eV
-2
-1
0
1
2
(-2)

(a)

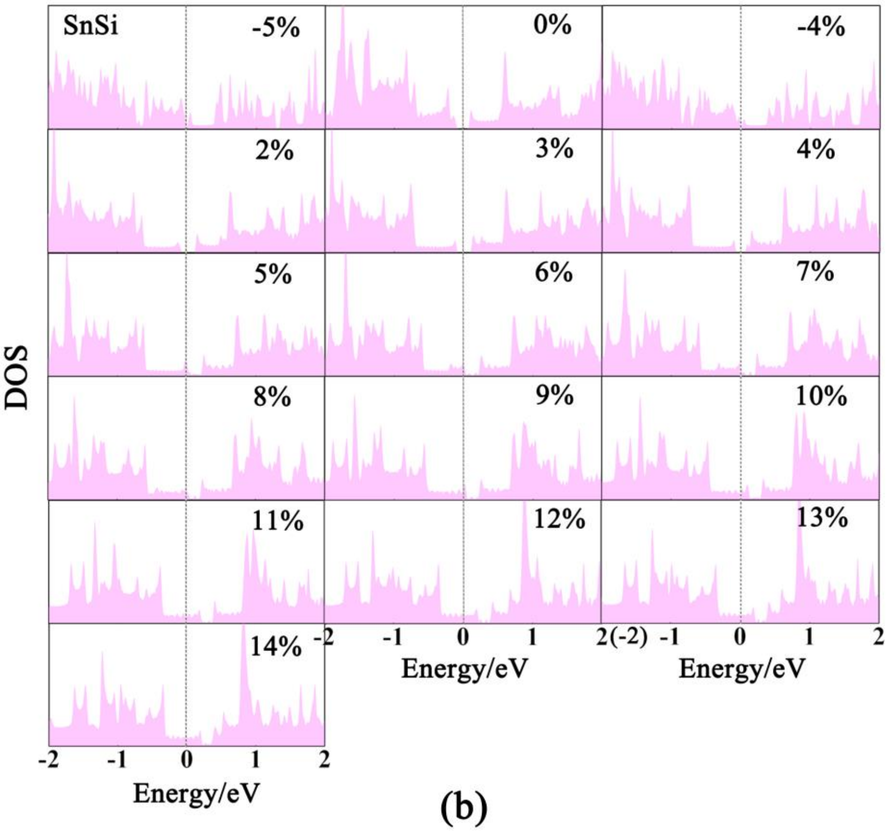
SnSi
-5%
0%
-4%
2%
3%
4%
5%
6%
7%
8%
9%
10%
11%
12%
13%
14%
DOS
Energy/eV
-2
-1
0
1
2
(-2)

(b)

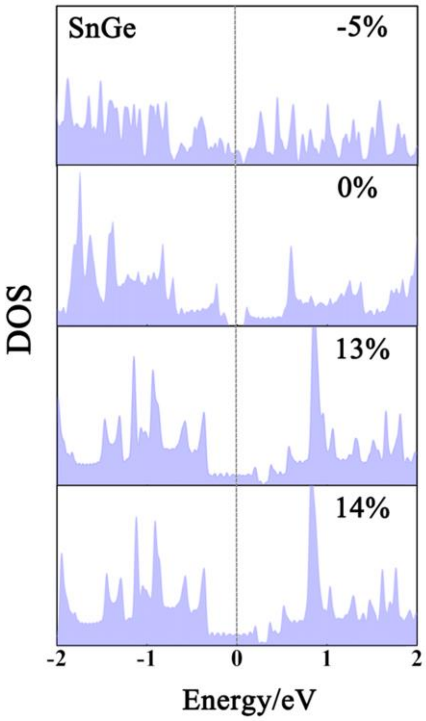

(c)

Figure S1 The computed DOSs of GeSi (a), SnSi (b) and SnGe (c) under biaxial strains where the computed ΔG_{H^*} values are ranging from -0.250 to 0.250 eV. For comparison, the computed DOSs of pristine GeSi, SnSi and SnGe monolayers are plotted.

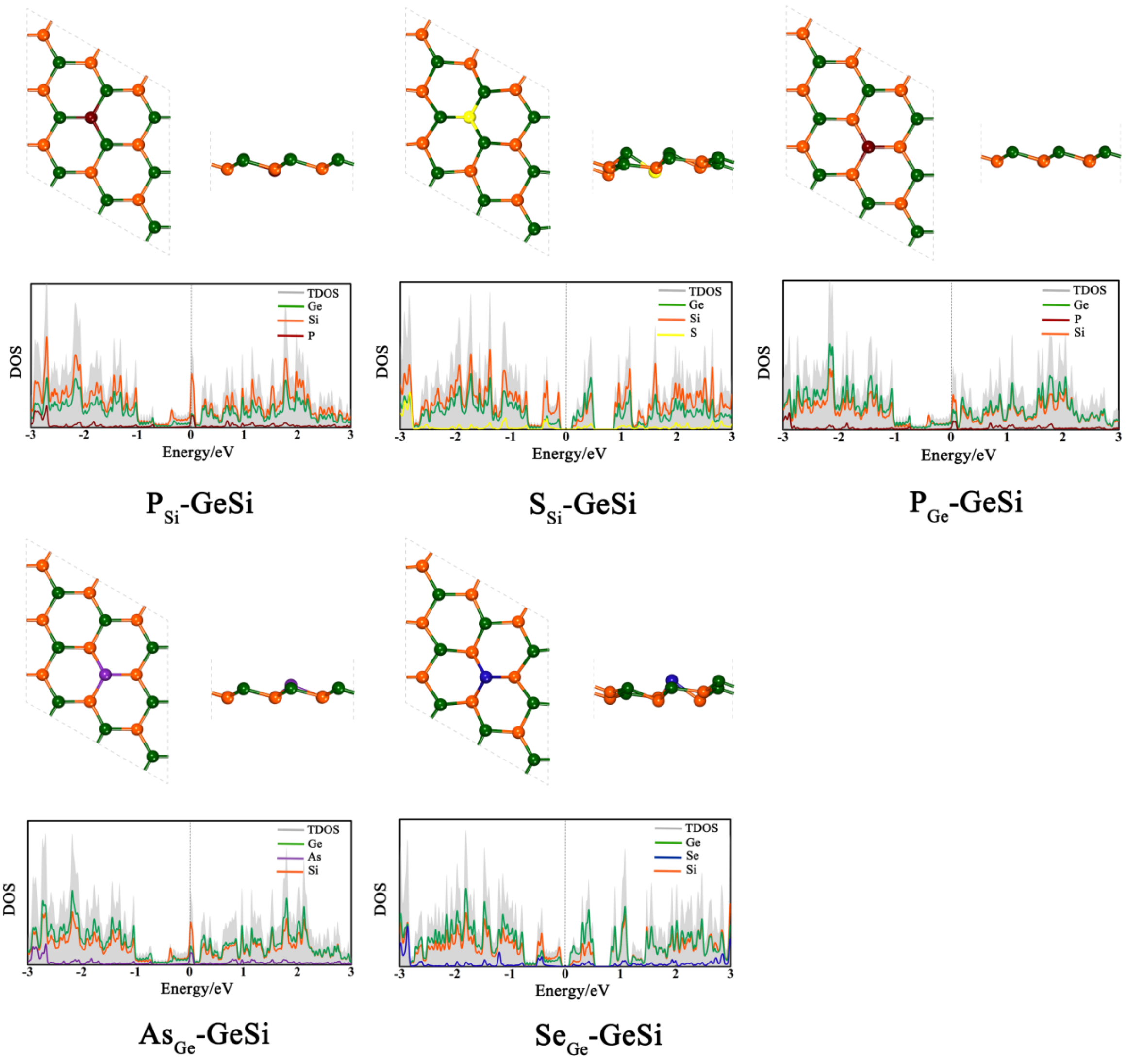

Figure S2 The top and side views of the doped GeSi structures and the corresponding DOSs.

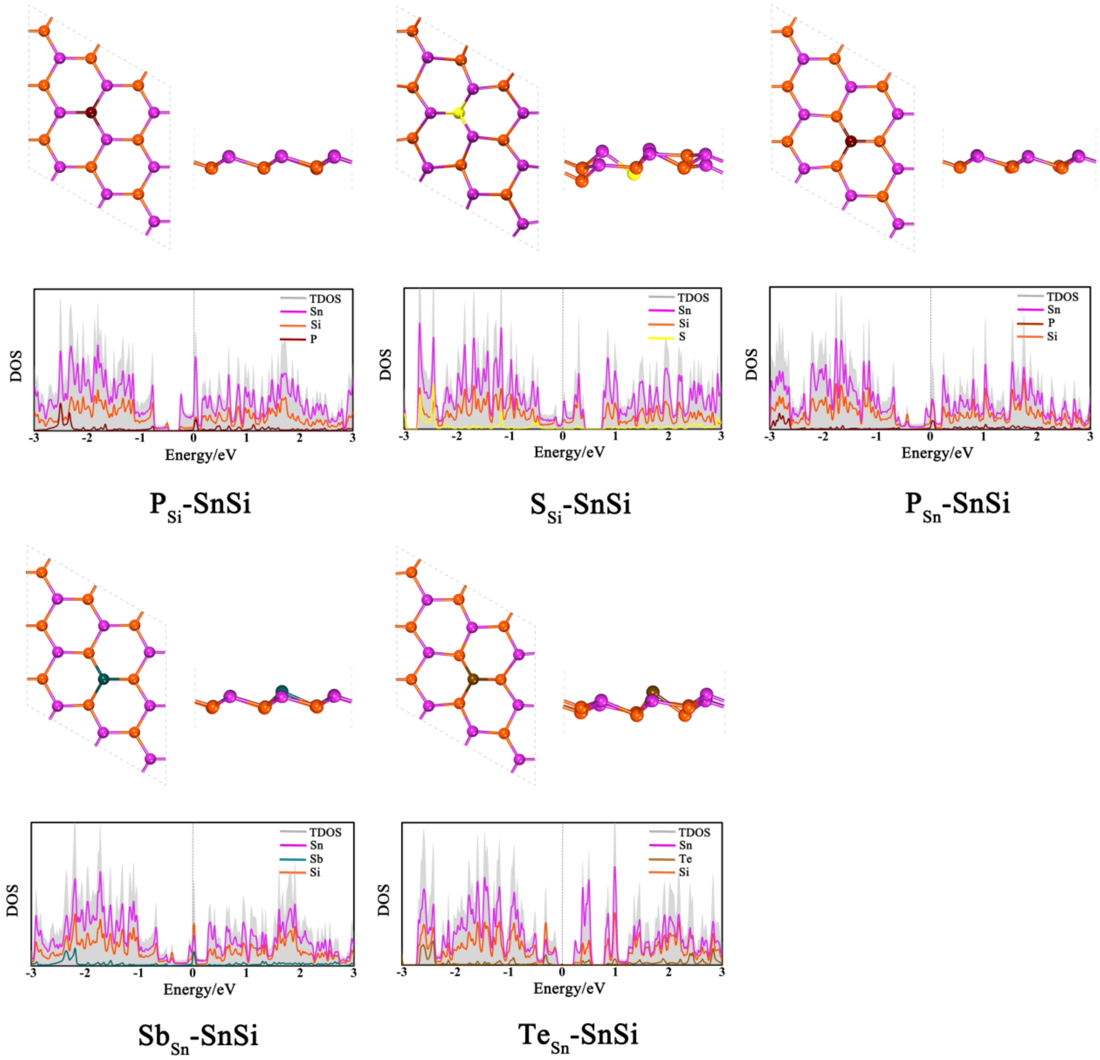

Figure S3 The top and side views of the doped SnSi systems and the corresponding DOSs.

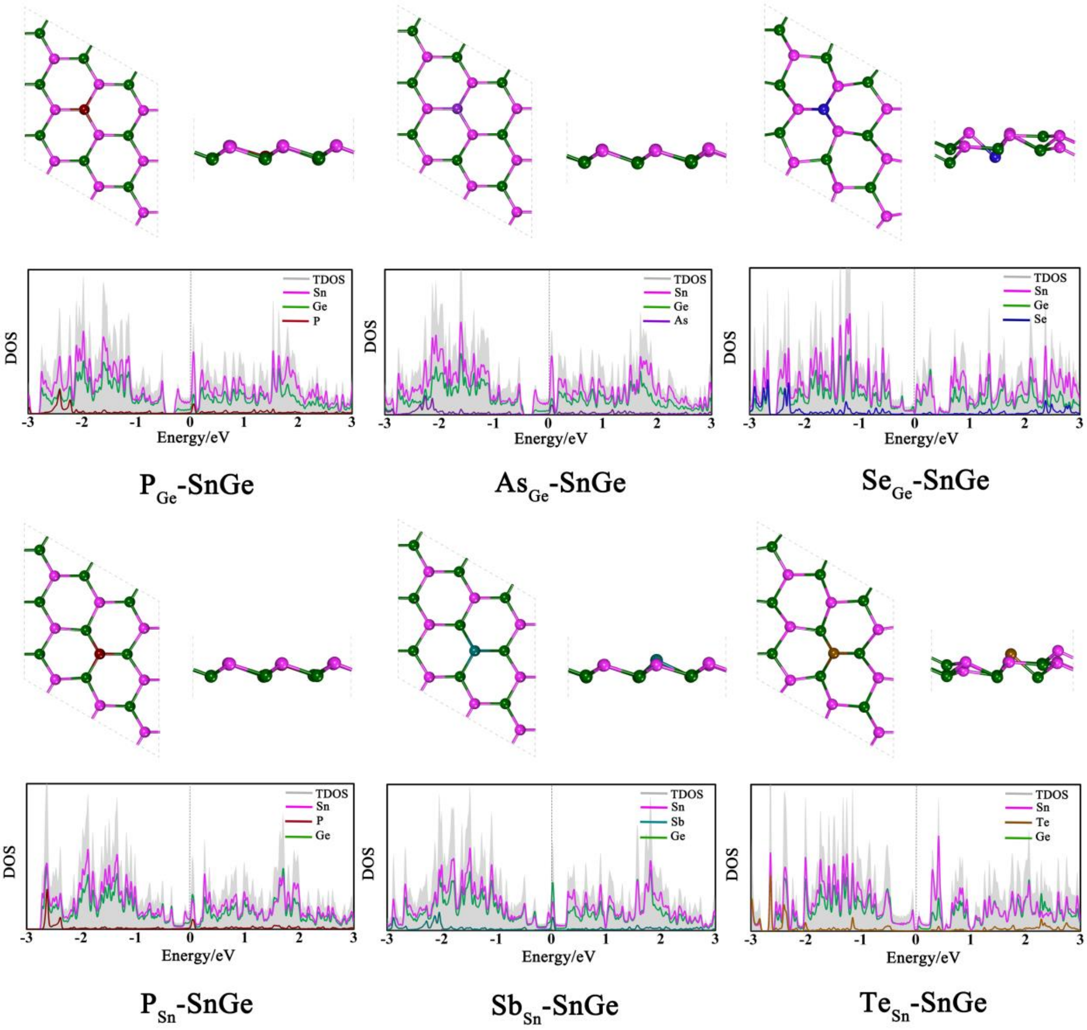

Figure S4 The top and side views of the doped SnGe structures and the corresponding DOSs.

Table S1 The space group, optimized lattice constant, the computed bond lengths d (Å), band gaps (eV) and elastic constants ($N.m^{-1}$) for 2D GeSi, SnSi and SnGe systems.

Systems	Space group	Lattice constant	d (Å)	Band gaps (eV)	Elastic constants C_{ij}, $N.m^{-1}$		
		a = b (Å)			C_{11}	C_{12}	C_{44}
GeSi	*P3M1*	3.926	2.345	Metallicity	59.41	17.71	20.92
SnSi	*P3M1*	4.234	2.547	0.249	15.76	10.29	12.06
SnGe	*P3M1*	4.305	2.610	0.213	31.58	17.05	8.10

Table S2 The computed ΔG_{H^*} (eV) values for H* at the different adsorption sites (S_{ad}) on the pristine GeSi, SnSi and SnGe systems.

Systems	S_{ad}	ΔG_{H^*} (eV)
GeSi	T_{Ge}	0.704
	T_{Si}	0.332
SnSi	T_{Sn}	0.826
	T_{Si}	0.293
SnGe	T_{Sn}	0.819
	T_{Ge}	0.620
Graphene	T_C	1.804

Table S3 The computed ΔG_{H^*} values at the T_{Ge}/T_{Si}, T_{Sn}/T_{Si} and T_{Sn}/T_{Ge} sites, as a function of compressive and tensile biaxial strains for the 2D GeSi, SnSi and SnGe.

	GeSi, ΔG_{H^*} (eV)		SnSi, ΔG_{H^*} (eV)		SnGe, ΔG_{H^*} (eV)	
Biaxial strain	T_{Ge}	T_{Si}	T_{Sn}	T_{Si}	T_{Sn}	T_{Ge}
-5%	0.460	0.084	0.631	-0.032	0.473	0.033
-4%	0.465	0.107	0.692	0.114	0.524	0.337
-3%	0.687	0.250	0.698	0.314	0.570	0.462
-2%	0.701	0.330	0.817	0.307	0.640	0.652
-1%	0.706	0.329	0.838	0.301	0.762	0.641
1%	0.704	0.321	0.836	0.276	0.829	0.621
2%	0.687	0.306	0.814	0.250	0.824	0.599
3%	0.668	0.287	0.807	0.244	0.820	0.576
4%	0.652	0.273	0.793	0.226	0.816	0.561
5%	0.634	0.250	0.783	0.201	0.787	0.537
6%	0.617	0.249	0.767	0.179	0.787	0.527
7%	0.595	0.228	0.762	0.148	0.768	0.498
8%	0.577	0.204	0.745	0.104	0.727	0.456
9%	0.555	0.184	0.720	0.029	0.732	0.388
10%	0.511	0.112	0.709	-0.015	0.704	0.344
11%	0.465	0.067	0.658	-0.068	0.674	0.300
12%	0.425	0.030	0.647	-0.134	0.646	0.262
13%	0.413	0.008	0.628	-0.182	0.627	0.228
14%	0.387	-0.054	0.617	-0.217	0.603	0.183

Table S4 The calculated band gaps for the GeSi, SnSi and SnGe as a function of compressive and tensible biaxial strains.

Biaxial strain	GeSi, band gap (eV)	SnSi, band gap (eV)	SnGe, band gap (eV)
-5%	0.076	0.049	Metallicity
-4%	Metallicity	0.057	0.098
-3%	Metallicity	0.223	0.095
-2%	Metallicity	0.241	0.061
-1%	Metallicity	0.258	0.233
0%	Metallicity	0.231	0.216
1%	Metallicity	0.251	0.189
2%	Metallicity	0.233	0.107
3%	Metallicity	0.241	0.069
4%	Metallicity	0.173	Metallicity
5%	Metallicity	Metallicity	Metallicity
6%	Metallicity	Metallicity	Metallicity
7%	Metallicity	Metallicity	Metallicity
8%	Metallicity	Metallicity	Metallicity
9%	Metallicity	Metallicity	Metallicity
10%	Metallicity	Metallicity	Metallicity
11%	Metallicity	Metallicity	Metallicity
12%	Metallicity	Metallicity	Metallicity
13%	Metallicity	Metallicity	Metallicity
14%	Metallicity	Metallicity	Metallicity

Table S5 The calculated ΔG_{H^*} values for the different adsorption sites on the X_{Si}-GeSi and X_{Ge}-GeSi (X = P, S As or Se) systems. Refer to Figure 5a-b for the different adsorption sites (S_{ad}).

S_{ad}	ΔG_{H^*} (eV)		S_{ad}	ΔG_{H^*} (eV)		
	P_{Si}-GeSi	S_{Si}-GeSi		P_{Ge}-GeSi	As_{Ge}-GeSi	Se_{Ge}-GeSi
T_{X1}	-0.055	1.085	T_{X1}	0.269	0.541	1.563
T_{Si2}	-0.093	0.217	T_{Si2}	-0.044	-0.021	0.273
T_{Si3}	-0.165	0.087	T_{Si3}	-0.039	-0.014	0.276
T_{Si4}	-0.171	0.097	T_{Si4}	-0.307	-0.327	-0.138
T_{Si5}	-0.170	0.079	T_{Si5}	-0.157	-0.139	0.209
T_{Si6}	-0.126	0.274	T_{Si6}	-0.301	-0.334	-0.137
T_{Ge7}	0.121	0.652	T_{Si7}	-0.151	-0.136	0.213
T_{Ge8}	-0.051	0.285	T_{Ge8}	0.405	0.440	0.653
T_{Ge9}	-0.046	0.284	T_{Ge9}	0.400	0.409	0.520
T_{Ge10}	0.120	0.661	T_{Ge10}	0.385	0.410	0.520
T_{Ge11}	0.240	0.684	T_{Ge11}	0.384	0.405	0.369
T_{Ge12}	0.239	0.686	T_{Ge12}	0.443	0.494	0.635

Table S6 The calculated ΔG_{H^*} values for the different adsorption sites on the X_{Si}-SnSi and X_{Sn}-SnSi (X = P, S, Sb or Te) systems. Refer to Figure 5c-d for the different adsorption sites (S_{ad}).

S_{ad}	ΔG_{H^*} (eV)		S_{ad}	ΔG_{H^*} (eV)		
	P_{Si}-SnSi	S_{Si}-SnSi		P_{Sn}-SnSi	Sb_{Sn}-SnSi	Te_{Sn}-SnSi
T_{X1}	0.023	0.853	T_{X1}	0.223	0.570	1.627
T_{Si2}	0.069	0.235	T_{Si2}	0.063	0.102	---
T_{Si3}	-0.067	-0.011	T_{Si3}	0.056	0.105	---
T_{Si4}	-0.099	-0.016	T_{Si4}	-0.231	-0.284	-0.013
T_{Si5}	-0.075	-0.006	T_{Si5}	-0.101	-0.081	0.198
T_{Si6}	0.044	0.170	T_{Si6}	-0.234	-0.286	-0.011
T_{Sn7}	0.296	0.785	T_{Si7}	-0.094	-0.077	0.214
T_{Sn8}	0.174	0.441	T_{Sn8}	0.491	0.519	0.730
T_{Sn9}	0.184	0.445	T_{Sn9}	0.491	0.511	0.645
T_{Sn10}	0.298	0.786	T_{Sn10}	0.483	0.510	0.575
T_{Sn11}	0.387	0.704	T_{Sn11}	0.486	0.515	0.662
T_{Sn12}	0.378	0.719	T_{Sn12}	0.508	0.562	---

Table S7 The calculated ΔG_{H^*} values for the different adsorption sites on the X_{Ge}-SnGe and X_{Sn}-SnGe (X = P, As, Se, Sb or Te) systems. Refer to Figure 5e-f for the different adsorption sites (S_{ad}).

S_{ad}	ΔG_{H^*} (eV)			S_{ad}	ΔG_{H^*} (eV)		
	P_{Ge}-SnGe	As_{Ge}-SnGe	Se_{Ge}-SnGe		P_{Sn}-SnGe	Sb_{Sn}-SnGe	Te_{Sn}-SnGe
T_{X1}	0.022	0.325	1.244	T_{X1}	0.017	0.478	1.472
T_{Ge2}	0.378	0.436	0.676	T_{Ge2}	0.412	0.448	0.089
T_{Ge3}	0.312	0.259	0.324	T_{Ge3}	0.424	0.441	0.122
T_{Ge4}	0.306	0.240	0.329	T_{Ge4}	0.148	0.099	0.410
T_{Ge5}	0.276	0.231	0.330	T_{Ge5}	0.282	0.288	-0.307
T_{Ge6}	0.411	0.410	0.467	T_{Ge6}	0.157	0.104	0.436
T_{Sn7}	0.296	0.306	0.672	T_{Ge7}	0.277	0.280	-0.302
T_{Sn8}	0.220	0.199	0.520	T_{Sn8}	0.453	0.475	0.669
T_{Sn9}	0.204	0.194	0.528	T_{Sn9}	0.460	0.479	0.504
T_{Sn10}	0.304	0.302	---	T_{Sn10}	0.471	0.478	0.392
T_{Sn11}	0.376	0.383	0.636	T_{Sn11}	0.464	0.473	0.507
T_{Sn12}	0.374	0.381	0.630	T_{Sn12}	0.490	0.526	0.374
